# Adaptation of *Globodera pallida* to Individual Components Compromises the Durability of Pyramided Resistance in Potato

**DOI:** 10.1111/eva.70181

**Published:** 2025-11-18

**Authors:** Océane Lechevalier, Magali Esquibet, Eric Grenier, Sylvain Fournet, Josselin Montarry

**Affiliations:** ^1^ IGEPP, INRAE Institut Agro, Univ Rennes Le Rheu France

**Keywords:** adaptation, evolutionary stepping stone, genome scan, *Globodera pallida*, pyramidal resistance, selection

## Abstract

The use of resistant plants is an effective alternative to chemical products. But their sustainability is often compromised by the rapid adaptation of pathogen populations. For the cyst nematode *Globodera pallida*, a major parasite of potato, several quantitative trait loci (QTLs) conferring resistance have been identified, but their individual use could lead to resistance breakdown. Combining several resistance loci within a single potato genotype has been proposed as a strategy to improve both efficacy and durability. However, the evolutionary pathways leading to the circumvention of this pyramidal resistance remain unknown. The combination of experimental evolution, phenotyping and genome scan enabled us to study the genomic basis of *G. pallida* adaptation to individual (*GpaV*_*spl*_, *GpaXI*_*spl*_) and pyramidal (*GpaV + XI*_*spl*_) resistance QTLs. Experimental evolution over 10 generations revealed that adaptation to *GpaV + XI*_*spl*_ pyramidal resistance was more difficult than to individual QTLs, but was nevertheless possible. Genomic analyses identified distinct regions under selection for each resistance, with a strong overlap between the adaptation to *GpaV*_*spl*_ and to *GpaV + XI*_*spl*_, but a weaker overlap between the adaptation to *GpaXI*_*spl*_ and to the pyramidal resistance. Known effector genes involved in immune suppression were systematically found in the selected regions, confirming their potential role in virulence. In addition, a two‐generations experiment demonstrated that prior adaptation, particularly to *GpaV*_*spl*_, facilitated adaptation to pyramidal resistance. These results highlight the existence of preferential evolutionary trajectories favored by genomic proximity between nematode lineages adapted to different resistances. Our results show that pyramidal resistance can be compromised by the prior deployment of its individual components, and underline the importance of taking evolutionary pathways into account in resistance deployment.

## 1 Introduction

The deployment of plant resistance genes represents a crucial alternative to chemical control in crop protection. In a context where pesticide use has a negative impact on the environment and human health, resistance genes offer a key alternative (Stuthman et al. 2007). However, despite their initial efficacy, many factors of resistance have shown limited durability. Combined with the repeated use of monocultures, this can lead to the emergence of virulent pathogen populations that overcome resistance (McDonald and Linde 2002). Rapid adaptation of pathogen populations has been repeatedly observed in various crop–parasite systems, including viruses, fungi, oomycetes, bacteria, and nematodes, both in the field and under experimental conditions (Fournet et al. 2013; García‐Arenal and McDonald 2003; Parlevliet 2002). These observations underline the urgent need to develop resistance strategies that are not only effective but also sustainable, especially considering the time and cost required to develop and deploy new resistance factors (Gilligan 2008).

To increase the durability of genetic resistance, different deployment strategies can be used, like mosaics (at the landscape scale), varietal mixtures and rotations (at the field scale) and gene pyramiding (at the plant scale). Mosaic deployment strategies, that is different cultivars in different fields of a continuous landscape, exploit evolutionary mechanisms by imposing selection on pathogens (Burdon et al. 2016; McDonald and Linde 2002; Zhan et al. 2015). Crop rotations, for example the succession of different cultivars in the same field (Curl 1963), can help limit pathogen adaptation, but their effectiveness depends on what the cultivars used are and how the cultivars are alternated over time. Among these strategies, gene pyramiding, which consists of combining several resistance loci within a single genotype, has proved particularly promising (Fuchs 2017). Theoretical studies and experimental works suggest that the pyramiding of resistance loci is more efficient and more difficult to overcome than individual QTLs, as it imposes stronger and more complex selective constraints on the parasite (Pilet‐Nayel et al. 2017; Rimbaud et al. 2018). The efficacy of pyramidal resistance has already been demonstrated several times in the case of cyst nematodes, with studies showing increased protection compared to single resistance loci (Caromel et al. 2005; Dalton et al. 2013; Meinhardt et al. 2021; Rigney et al. 2017; Rouppe van der Voort et al. 2000). However, if a virulent pathogen capable of overcoming all resistance loci emerges, the break is complete, leading to the loss of all resistance components at once. It is therefore crucial to better understand the evolutionary pathways that could lead to such an adaptation.

The potato cyst nematode *Globodera pallida* poses a major threat to potato production. In Europe, *G. pallida* management has largely relied on the deployment of resistance derived from the wild *Solanum vernei* species and attributed to the major QTL *GpaV*_*vrn*_. However, field observations and experimental evolution studies have shown that *G. pallida* populations can adapt to this resistance after several generations, raising concerns about its durability (Fournet et al. 2013; Mwangi et al. 2019; Niere et al. 2014). In this context, the identification of alternative sources of resistance is becoming necessary. One of these alternatives is another source of resistance from *Solanum sparsipilum*, which harbors the major QTL *GpaV*_*spl*_. This QTL represents a promising option, particularly as a recent study has shown that there is no cross‐virulence between *G. pallida* populations adapted to resistance conferred by *GpaV*_*vrn*_ and those adapted to *GpaV*_*spl*_ resistance (Lechevalier et al. 2025). This suggests that *GpaV*_*spl*_ could be an effective solution for controlling *GpaV*_*vrn*_ adapted *G. pallida* populations. However, adaptation to *GpaV*_*spl*_ was also shown to be possible in experimental evolution, as rapidly as that to *GpaV*_*vrn*_, underlining the need for more robust strategies (Lechevalier et al. 2025). To improve both efficacy and durability, the combination of *GpaV*_*spl*_ with another minor resistance QTL, *GpaXI*_*spl*_, was explored. The resulting pyramidal resistance, *GpaV + XI*_*spl*_, has already demonstrated strong efficacy and provides a relevant model for assessing the benefits and limitations of resistance factor pyramiding (Caromel et al. 2005).

*Globodera pallida* is a sexually reproducing diploid endoparasite. After hatching in the soil, second‐stage juveniles (J2) enter the roots of the host plant. During this process, J2 secrete effectors via their stylet into plant cells, modulating host cell functions to allow the formation of a specialized feeding structure called syncytium (Ali et al. 2017; Eves‐van den Akker 2021; Mitchum et al. 2013). The quality of the syncytium largely determines the nematode's trajectory of differentiation into male or female (Sobczak and Golinowski 2011). At feeding sites where conditions are optimal, juveniles develop into sedentary females and then into cysts. Conversely, less well‐established syncytia generally lead to the development of mobile males that can leave the root system to reproduce with females. The cycle generally lasts several weeks and results in the formation of cysts containing hundreds of eggs that can persist in the soil for years, making control of this pest particularly difficult (Evans and Stone 1977).

Recent advances in population genomics of plant‐parasitic nematodes now offer powerful tools to investigate the molecular basis of adaptation to plant resistance (Montarry et al. 2021). By combining genomic data with experimental evolution, it becomes possible to identify the genomic regions involved in virulence and to trace the evolutionary trajectories leading to resistance breakdowns (e.g., Lechevalier et al. 2025). The present study aimed to investigate the genomic basis of adaptation to individual QTLs (*GpaV*_*spl*_ or *GpaXI*_*spl*_) and to the combination of both QTLs (*GpaV + XI*_*spl*_). We combined experimental evolution, phenotyping and genome scan analyses to identify genomic regions associated with virulence and to assess whether shared selection signatures exist between individual and combined resistances. Moreover, we phenotypically tested whether prior adaptation to an individual QTL could serve as a stepping stone for adaptation to pyramidal resistance.

## 2 Materials and Methods

### 2.1 Plant Material and Experimental Evolution Design

The initial population was derived from *Globodera pallida* cysts originating from an infested field near Saint‐Malo (France). The experimental evolution was designed using different potato genotypes, all originating from a cross between a diploid clone spl329.18 of the *Solanum sparsipilum* (the resistant parent) and Caspar H3 (the susceptible parent), a dihaploid of *Solanum tuberosum* cv. Caspar (Caromel et al. 2005). Four potato genotypes were used to rear nematode lineages: 96D31.69 (no resistant QTL), 96D31.51 (harboring the resistant QTL *GpaV*_*spl*_), 96D31.137 (harboring *GpaXI*_*spl*_), and 96D31.132 (harboring both *GpaV*_*spl*_ and *GpaXI*_*spl*_). Presence or absence of both QTLs was followed using the molecular markers ASC151 for *GpaV*_*spl*_ and MS137 and C2_At5g60600 for *GpaXI*_*spl*_ (unpublished markers derived from the QTL fine‐mapping study of Caromel et al. (2005) and from Kerlan et al. (2016)). These genotypes differ in their resistance mechanisms: *GpaV*_*spl*_ acts as a masculinizing resistance, while the *GpaV + XI*_*spl*_ combination confers blocking resistance (Caromel et al. 2005). Independent replicate lineages were established on each host genotype, with two replicates on 96D31.69, three on 96D31.51, two on 96D31.137, and five on 96D31.132. Cysts were extracted after each generation and used to infect plants of the following generation until 10 generations were obtained under greenhouse conditions (see Supplementary Methods for details). Given that *G. pallida* completes one generation per year under European conditions, this corresponds to a 10‐year experiment.

To test whether prior adaptation to *GpaV*_*spl*_ or to *GpaXI*_*spl*_ could facilitate adaptation to the pyramided *GpaV + XI*_*spl*_ resistance, a short experimental evolution was set up. Lineages from the tenth generation of the previous experimental evolution were reared on 96D31.132 (*GpaV + XI*_*spl*_) for two successive generations starting from 50 cysts (Methods S1).

### 2.2 Phenotyping

Three phenotyping experiments were conducted on the *G. pallida* lineages obtained through experimental evolutions. All experiments followed the standardized Petri dish protocol described by Fournet et al. (2013). The first experiment aimed to quantify the virulence level of each lineage, obtained after 10 generations of the experimental evolution (G10), on their specific potato genotype: 96D31.69 (susceptible), 96D31.51 (*GpaV*_*spl*_), 96D31.137 (*GpaXI*_*spl*_), and 96D31.132 (*GpaV + XI*_*spl*_). The second experiment was designed to test the hypothesis that preadaptation to one resistance source (*GpaV*_*spl*_ or *GpaXI*_*spl*_) could facilitate adaptation to the combined resistance (*GpaV + XI*_*spl*_). To this end, all G10 lineages were inoculated onto genotype 96D31.132 (*GpaV + XI*_*spl*_). The third experiment evaluated the impact of a short evolutionary stepping stone on lineages from G10. The G10 lineages were subjected to two successive generations on 96D31.132. The resulting G2TE lineages were then phenotyped on 96D31.132.

For all experiments, second‐stage juveniles (J2) were obtained by hatching cysts in root exudates from the susceptible cv. Désirée. Newly hatched J2 were inoculated onto potato roots in Petri dishes, and the number of females was counted after 26 days of development. Statistical analyses were performed using the R software version 4.2.2. For each potato genotype, the lineage effect was tested for the female count using a one‐way ANOVA (see Methods S1 for details). Multiple comparisons of means were performed using the Tukey test (α = 0.05).

### 2.3 Extraction, Library Preparation and Sequencing

To perform pool‐seq, eight pools were created for each experimental lineage (i.e., 4 potato cultivars (96D31.69, 96D31.51, 96D31.137, 96D31.132) × 2 replicates). The optimal size of each pool was determined using the PIF tool, which assesses the accuracy of allele frequency estimation from pool‐based NGS population data (Gautier et al. 2013). Each pool consisted of 600 diploids J2 stage juvenile larvae from 200 distinct cysts (i.e., 3 J2 per cyst). DNA was extracted directly from each pool using the Qiagen DNeasy Blood and Tissue Kit (Qiagen, Hilden, Germany) following the manufacturer's instructions. Low Pass Sequencing was performed at the GeT‐PlaGe core facility (INRAE Toulouse, France) (Methods S1). DNA quality controls were performed using the Qubit 2.0 Fluorometer and the NanoDrop 8000 Spectrophotometer, estimating an average DNA quantity of 68 ng per pool before library preparation. DNA was sequenced on one S4 NovaSeq 6000 lane with a paired‐end read length of 2 × 150 bp using the Illumina S4 Reagent Kit v1.5 (300 cycles) (20028312). All raw sequencing files generated have been deposited in the NCBI Sequence Read Archive (doi to be completed).

### 2.4 Processing of Pool Seq Data

After quality control and filtering, reads were mapped against the *G. pallida* D383 reference genome (van Steenbrugge et al. 2023), which consists of 163 scaffolds and spans approximately 113 Mb. Mapping was performed using the MEM algorithm from BWA v0.7.17 (Li 2013) with default settings (Methods S1). The resulting BAM files were sorted and duplicates removed using *Picard* tool v2.18.2 (broadinstitute.github.io/picard/). Variant calling was performed using the haplotype caller implemented in *FreeBayes* v1.1.0 (Garrison and Marth 2012) with the following options ‐K ‐C 1 ‐F 0.01 ‐G 5 ‐n 4 ‐m 30 ‐q 20. The resulting vcf file was parsed with the *vcf2pooldata* function of the R package *poolfstat* (v2.2.0) (Gautier et al. 2022) with options min.maf = 0.01, min.cov.per.pool = 20 and max.cov.per.pool = 400.

### 2.5 Detection and Content of Regions Involved in Adaptations

Genome scans to detect signatures of selection and associations with virulence were performed using *BayPass* v2.41 (Gautier 2015). The method is an *F*_*ST*_‐based approach to evaluate associations between variation in phenotypic/ecological variables and genetic markers, and to identify candidate loci for adaptive divergence while controlling for neutral covariance of alleles across populations. Following the *BayPass* manual recommendation (see also Gautier et al. 2018), the complete dataset was first split into 31 subsamples of 75,000 SNPs. This subsampling strategy allows a more efficient analysis as it requires less computational time because each of the 31 pseudo‐independent files is run in parallel, while also reducing background linkage disequilibrium within each subset, which improves parameter estimation. The *C*_2_ statistic (Olazcuaga et al. 2020) implemented in *BayPass* was used to identify SNPs associated with virulence status, while accounting for population structure. Four analyses were carried out, each comparing avirulent lineages reared on 96D31.69 (No resistant QTL) with virulent lineages reared on 96D31.132 (*GpaV + XI*_*spl*_) or 96D31.51 (*GpaV*_*spl*_) or 96D31.137 (*GpaXI*_*spl*_). *C*_2_ estimates were carried out in three independent analyses (with different seeds) for each data comparison. As *C*_2_ estimates were very consistent across the three independent analyses, only the results of the first analysis have been reported. To identify and delimit the significant regions associated with virulent status, a local scoring approach described by Fariello et al. (2017) was performed based on the *C*_2_ values obtained. The local score approach is implemented in the R function compute.local.scores available in the *BayPass* software package (v3.0) and ran with default options. The analysis was run with default parameters, that is, xi = 1 and *pval.local.score.thres* = 0.01, as described in Fariello et al. 2017. In this framework, xi determines how signals from neighboring SNPs are accumulated and corresponds to the *p* value significance thresholds, and are therefore computed automatically by the function based on the *pval.local.score.thres* argument, which defines the proportion of the highest score values to retain (here the top 1%). SNPs located in top candidate regions identified by local score were extracted. Filters were applied to select SNPs with consistent selection signals, and further analysis was carried out to investigate their potential functional impact at the gene level, focusing on candidates potentially involved in suppressing host plant immunity (see details in Methods S1).

## 3 Results

### 3.1 Selection Effect and Resistance Effectiveness

**FIGURE 1:**
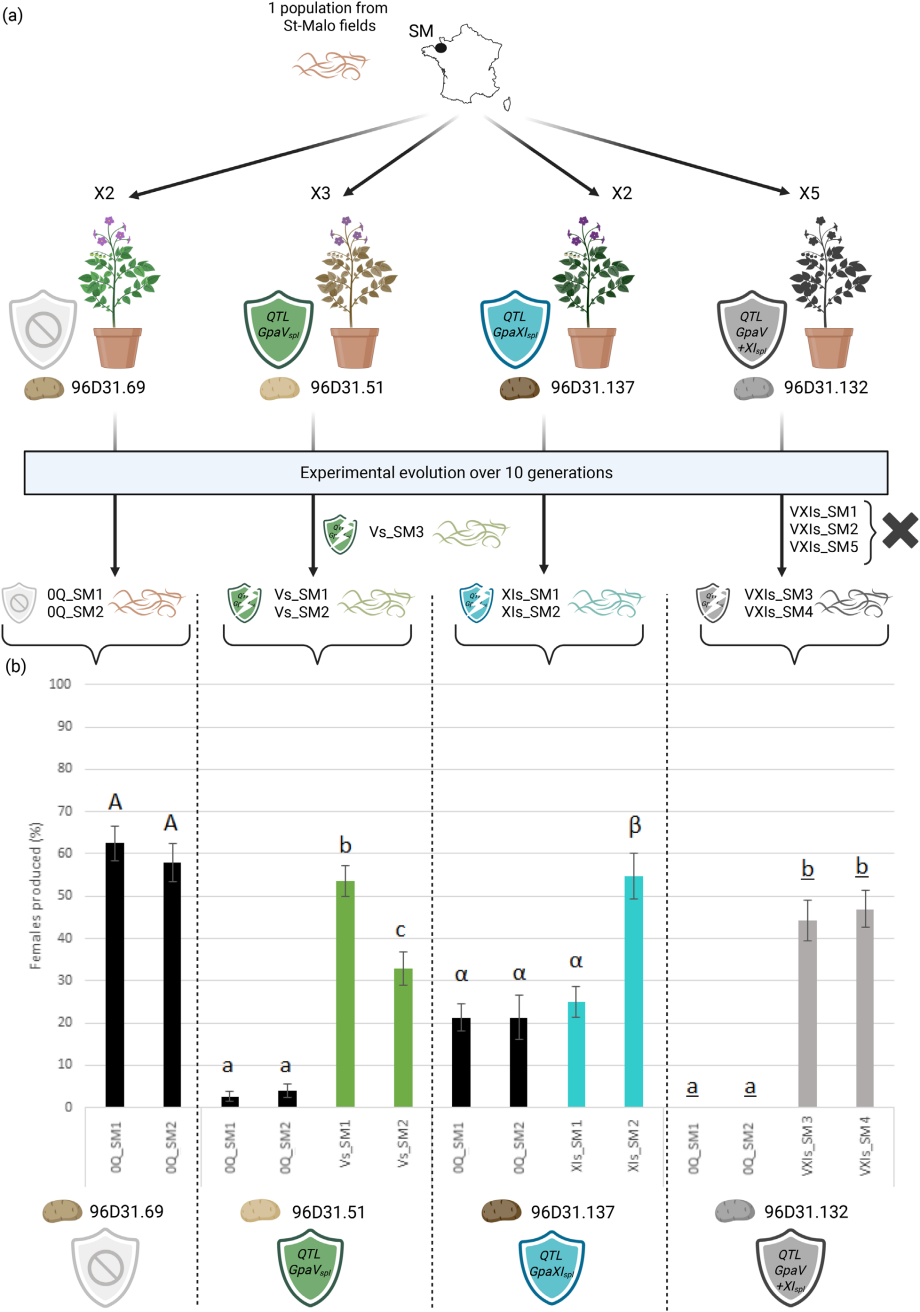
Experimental evolution and phenotyping of *Globodera pallida* lineages on susceptible and resistant potato genotypes. (a) Overview of the experimental evolution design. Twelve independent *G. pallida* lineages were initially established and reared for 10 generations (G10) on potato genotypes either susceptible (96D31.69: 0QTL) or carrying one or two resistance QTLs (96D31.51: *GpaV*_*spl*_; 96D31.137: *GpaXI*_*spl*_; 96D31.132: *GpaV + XI*_*spl*_). All lineages were successfully maintained on 0QTL, *GpaV*_*spl*_, and *GpaXI*_*spl*_ genotypes, while only two out of five lineages could be maintained for 10 generations on the pyramidal resistance *GpaV + XI*_*spl*_. (b) The resulting *G. pallida* lineages reared on *GpaV*_*spl*_ (Vs_SM1 and Vs_SM2), *GpaXI*_*spl*_ (XIs_SM1 and XIs_SM2), and *GpaV + XI*_*spl*_ (VXIs_SM3 and VXIs_SM4) were phenotyped on their respective resistant potato genotypes: 96D31.51, 96D31.137, and 96D31.132. Control lineages reared on the susceptible genotype 96D31.69 (0Q_SM1 and 0Q_SM2) were inoculated on the different potato genotypes to confirm the effectiveness of each resistance. The virulent status of the independent lineages was confirmed on their respective hosts, as they produced significantly more females than the control lineages.

Experimental evolution enabled us to obtain independent lineages reared for 10 generations on potatoes with or without resistance QTL. A total of 12 independent lineages were initially established: two on the susceptible genotype without resistance QTL 96D31.69 (0QTL), three on 96D31.51 (*GpaV*_*spl*_), two on 96D31.137 (*GpaXI*_*spl*_) and five on 96D31.132 (*GpaV + XI*_*spl*_). While all lineages were successfully maintained on genotypes 96D31.69, 96D31.51 and 96D31.137, the development on 96D31.132 was rare as only 40% of initial lineages on 96D31.132 were successfully maintained over 10 generations, while 100% of the other lineages were successfully maintained over 10 generations (Fisher's exact test *p* < 0.0001; Figure 1a).

The resulting tenth‐generation lineages (G10) were then phenotyped on their respective selection hosts to quantify their level of virulence (Figure 1b). Control lineages reared on the susceptible host 96D31.69 (0Q_SM1, 0Q_SM2) produced an average of 60.25% females on this host. The Vs_SM1 and Vs_SM2 lineages reared on 96D31.51 (*GpaV*_*spl*_) produced 53.5% and 32.87% females on this same genotype respectively, compared to only 3.34% on average for the 0Q_SM1 and 0Q_SM2 lineages (*F*_3,60_ = 70.94, *p* < 0.0001). Regarding the lineages reared on 96D31.137 (*GpaXI*_*spl*_), lineage XIs_SM2 produced 54.67% females on this same genotype, while the XIs_SM1 lineage produced as many females (25%) as the average produced by 0Q_SM1 and 0Q_SM2 (21.33%) (*F*_3,55_ = 12.57, *p* < 0.0001). Lineages VXIs_SM3 and VXIs_SM4 reared on the genotype 96D31.132 (*GpaV + XI*_*spl*_) produced an average of 45.61% females on this genotype, whereas no females were counted for the 0Q_SM1 and 0Q_SM2 lineages (*F*_3,55_ = 32.70, *p* < 0.0001). These results therefore demonstrated a gradient of resistance efficacy with *GpaXI*_*spl*_ conferring low resistance, *GpaV*_*spl*_ conferring high resistance and finally *GpaV + XI*_*spl*_ conferring very high resistance. After 10 generations of experimental evolution, the lineages selected for phenotyping were able to produce females on their respective resistance genotypes, whatever the initial resistance efficiency.

### 3.2 Pre‐Adaptation to Individual QTL to Overcome the Combined QTL

**FIGURE 2:**
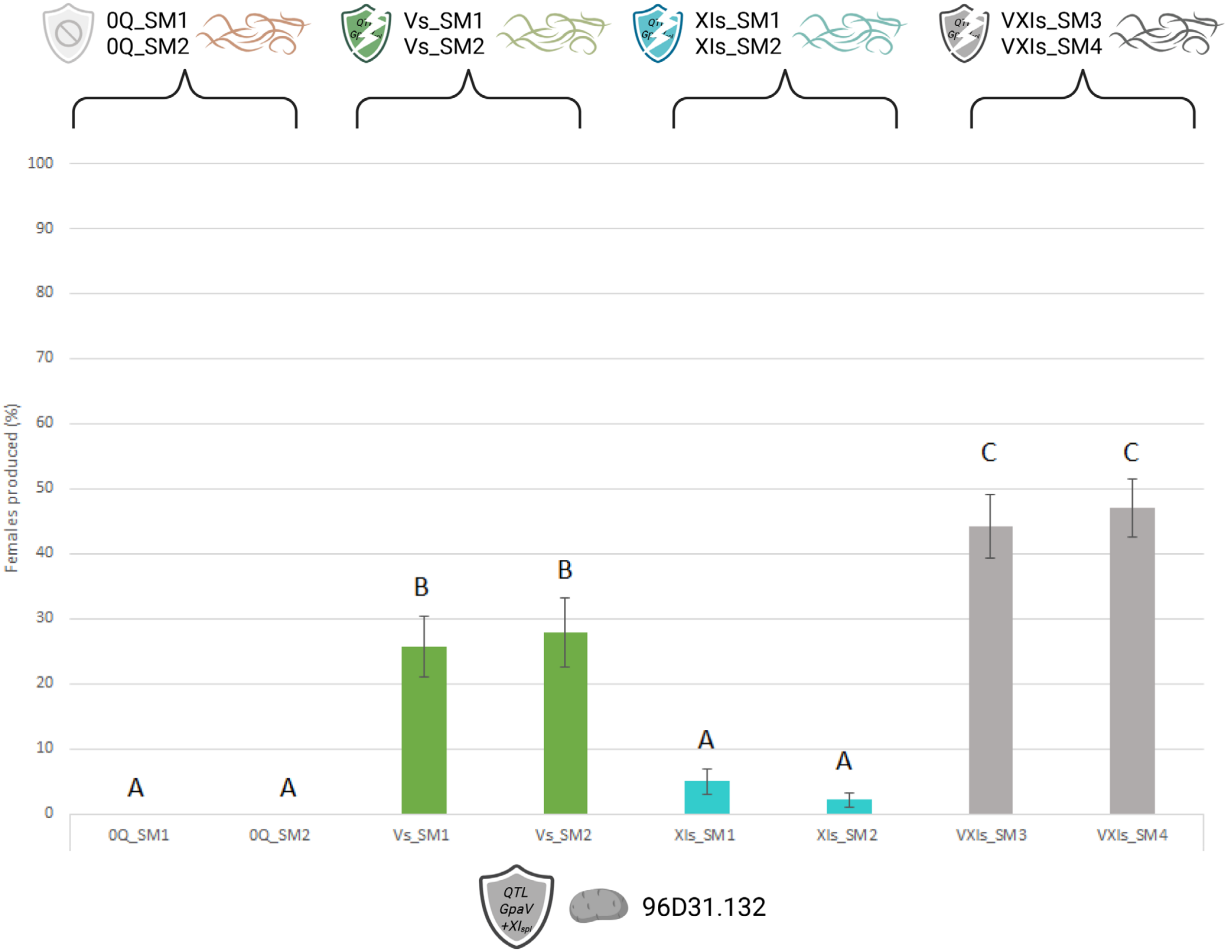
Phenotyping of lineages obtained by experimental evolution on the pyramided resistance genotype 96D31.132 (*GpaV + XI*_*spl*_). Percentage of females produced on potato genotype 96D31.132 (*GpaV + XI*_*spl*_) by eight nematode lineages: Two control lineages reared on susceptible host (0Q_SM1, 0Q_SM2), two lineages evolved on genotype 96D31.51 (*GpaV*_*spl*_) (Vs_SM1, Vs_SM2), two lineages evolved on genotype 96D31.37 (*GpaXI*_*spl*_) (XIs_SM1, XIs_SM2), and two lineages evolved directly on the combined genotype 96D31.32 (*GpaV + XI*_*spl*_) (VXIs_SM3, VXIs_SM4). Letters indicate statistically significant differences between groups (ANOVA, *F*_7,107_ = 24.29, *p* < 0.0001).

New phenotyping was designed to test whether preadaptation to the individual *GpaV*_*spl*_ or *GpaXI*_*spl*_ QTL could confer an advantage on adaptation to the *GpaV + XI*_*spl*_ combination. Where the control lineages did not produce any females on 96D31.132, all the lineages adapted to the individual QTLs were able to produce some (Figure 2). The Vs_SM1 and Vs_SM2 lineages reared on 96D31.51 (*GpaV*_*spl*_) produced an average of 26.79% females on the 96D31.132 (*GpaV + XI*_*spl*_) genotype, compared with an average of 3.58% for the XIs_SM1 and XIs_SM1 lineages reared on 96D31.137 (*GpaXI*_*spl*_). Despite their ability to partially overcome the combined resistance, the Vs and XIs lineages produced significantly fewer females than the VXIs_SM3 and VXIs_SM4 lineages, directly evolved on the combined genotype, which averaged 45.61% females. The differences between lineages were statistically significant (*F*_7,107_ = 24.29, *p* < 0.0001, Figure 2). These results indicate a more marked partial preadaptation via *GpaV*_*spl*_ than via *GpaXI*_*spl*_.

### 3.3 Genome Scan Comparison of Adaptation to Resistances

Sequencing was performed on eight pools, each composed of 600 diploid individuals. The sample set contained an average of 224 M sequencing reads, 88.47% of which were correctly aligned to the D383 reference genome. After applying filtering steps including duplicate removal, quality filtering, minimum coverage thresholds and indel exclusion, the sample set consisted of an average of 118 M reads. This filtering resulted in an average depth of approximately 156X on the 113 Mb genome. After variant calling, additional filtering steps were applied to ensure the quality and reliability of the SNP dataset. These steps included thresholds on coverage and minimum allele frequency in the pools. The resulting variant call file was then processed using *poolfstat*, leading to a final dataset of 2,378,173 SNPs.

**FIGURE 3:**
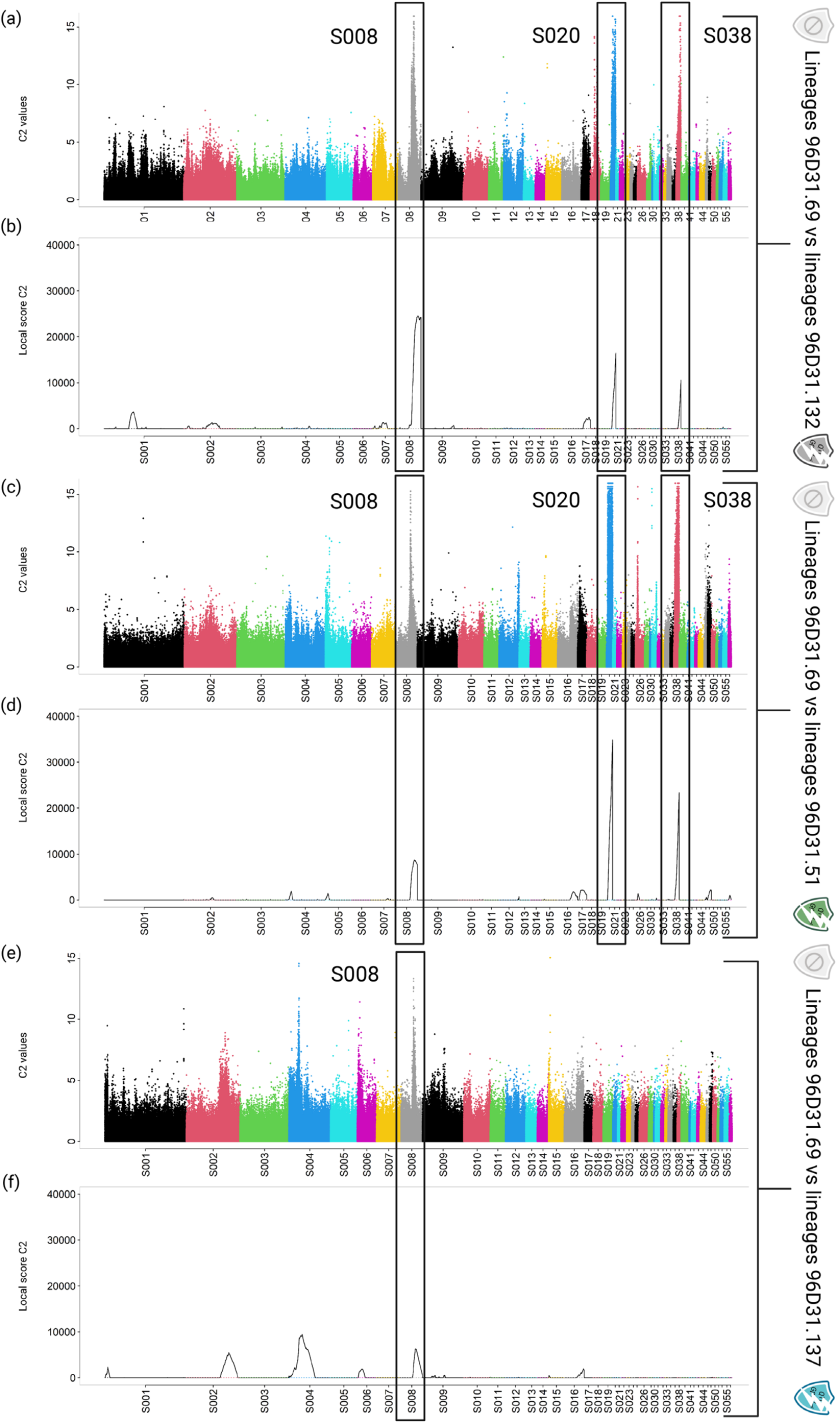
Manhattan plots showing selection signatures in the *Globodera pallida* genome detected between virulent and avirulent lineages. Manhattan plots (a) and (b) were constructed from SNPs derived from the comparison between lineages reared on 96D31.69 and 96D31.132 (i.e., adaptation to *GpaV + XI*_*spl*_), Manhattan plots (c) to (d) from the comparison between lineages 96D31.69 and 96D31.51 (i.e., adaptation to *GpaV*_*spl*_), and finally Manhattan plots (e) to (f) from the comparison between lines 96D31.69 and 96D31.137 (i.e., adaptation to *GpaXI*_*spl*_). Scaffold names are shown on the horizontal axis; the vertical axis shows for each SNP the *C*_2_ values for (a), (c) and (e), and the local *C*_2_ score values for (b), (d) and (f). SNPs with high *C*_2_ values and genomic regions with high local *C*_2_ score values are strongly associated with the virulence status. Boxes highlight scaffolds containing regions with strong indications of selection.

This dataset was used to perform genome scan analyses aiming to identify genomic regions under selection. In particular, the C2 statistic was used to detect SNPs significantly associated with virulence in each of the three comparisons investigated (Figure 3a,c,e). These analyses contrasted the two avirulent lineages reared on the susceptible 96D31.69 genotype (0Q_SM1 and 0Q_SM2) with (i) the two virulent lineages adapted to combined *GpaV + XI*_*spl*_ resistance (VXIs_SM3 and VXIs_SM4), (ii) the two virulent lineages adapted to *GpaV*_*spl*_ resistance (Vs_SM1 and Vs_SM2), and (iii) the two virulent lineages adapted to *GpaXI*_*spl*_ resistance (XIs_SM1 and XIs_SM2). To refine the detection of regions under selection, a local score analysis based on *C*_2_ values was performed (Figure 3b,d,f). This approach enabled the identification and delineation of genomic regions, thus improving the resolution of candidate signals. In the comparison involving combined resistance (*GpaV + XI*_*spl*_), three high candidate regions for selection were detected on scaffolds S008, S020 and S038. A similar pattern was observed in the comparison involving *GpaV*_*spl*_ resistance, although the signal on S008 was less pronounced. In contrast, for the comparison involving *GpaXI*_*spl*_ resistance only the weak signal on S008 was detected.

### 3.4 SNP Content Comparison of Candidate Regions

SNPs located in candidate genomic regions identified by local score *C*_2_ analysis were extracted to further characterize the genomic signature of adaptation. For each comparison, several hundred significant windows showed an accumulation of SNPs associated with virulence. In the comparison involving the combined QTL *GpaV + XI*_*spl*_ (VXIs_SM3 and VXIs_SM4 vs. 0Q_SM1 and 0Q_SM2), a total of 495 significant windows were identified. The three main regions are made up of: a large region of around 1.7 Mb on S008 (22,379 SNPs), a region of 625 kb on scaffold S020 (7313 SNPs) and a smaller region of 365 kb on scaffold S038 (5642 SNPs), making a total of over 35,334 SNPs (Table 1).

**TABLE 1:** Summary of the top three candidate genomic regions identified by local *C*_2_ score analysis for each resistance comparison.

Comparison	Scaffold	Scaffold size (bp)	Region start	Region end	Region size (bp)	Number of SNPs
*GpaV + XI*_*spl*_	S008	3,470,396	1,734,109	3,470,312	1,736,203	22,379
*GpaV*_*spl*_	S008	3,470,396	2,315,552	3,470,312	1,154,760	14,421
*GpaXI*_*spl*_	S008	3,470,396	1,952,792	3,470,312	1,517,520	18,091
*GpaV + XI*_*spl*_	S020	1,257,442	630,679	1,255,470	624,791	7313
*GpaV*_*spl*_	S020	1,257,442	624,659	1,255,470	630,811	9003
*GpaV + XI*_*spl*_	S038	1,036,861	672,458	1,036,711	364,253	5642
*GpaV*_*spl*_	S038	1,036,861	421,915	1,036,711	614,796	7997

*Note:* For each region, the scaffold and its size, total region size, region start and end coordinates, number of SNPs included are provided. Comparisons include *GpaV*_*spl*_ (Vs_SM1 and Vs_SM2 vs. 0Q_SM1 and 0Q_SM2), *GpaXI*_*spl*_ (XIs_SM1 and XIs_SM2 vs. 0Q_SM1 and 0Q_SM2), and *GpaV + XI*_*spl*_ (VXIs_SM3 and VXIs_SM4 vs. 0Q_SM1 and 0Q_SM2).

Comparison involving the individual QTL *GpaV*_*spl*_ (Vs_SM1 and Vs_SM2 vs. 0Q_SM1 and 0Q_SM2) returned 842 significant windows. The same three main scaffolds as for V + XI were involved, but with different extents and numbers of SNPs. The region on S008 spanned 1.15 Mb and contained 14,421 SNPs, fewer than for V + XI. The regions on S020 and S038 were of comparable size to those of *GpaV + XI*_*spl*_ (630 and 615 kb, respectively), with an equally comparable number of SNPs (9003 and 7997 SNPs, respectively). The three regions thus constituted a total of 31,421 SNPs.

The comparison involving the individual QTL *GpaXI*_*spl*_ (XIs_SM1 and XIs_SM2 vs. 0Q_SM1 and 0Q_SM2) resulted in 582 significant windows. In contrast to the other comparisons, only one large region was detected: a 1.5 Mb segment on S008, containing 18,091 SNPs. This number is closer to that observed in *GpaV + XI*_*spl*_ on S008, and significantly higher than in *GpaV*_*spl*_ alone.

**FIGURE 4:**
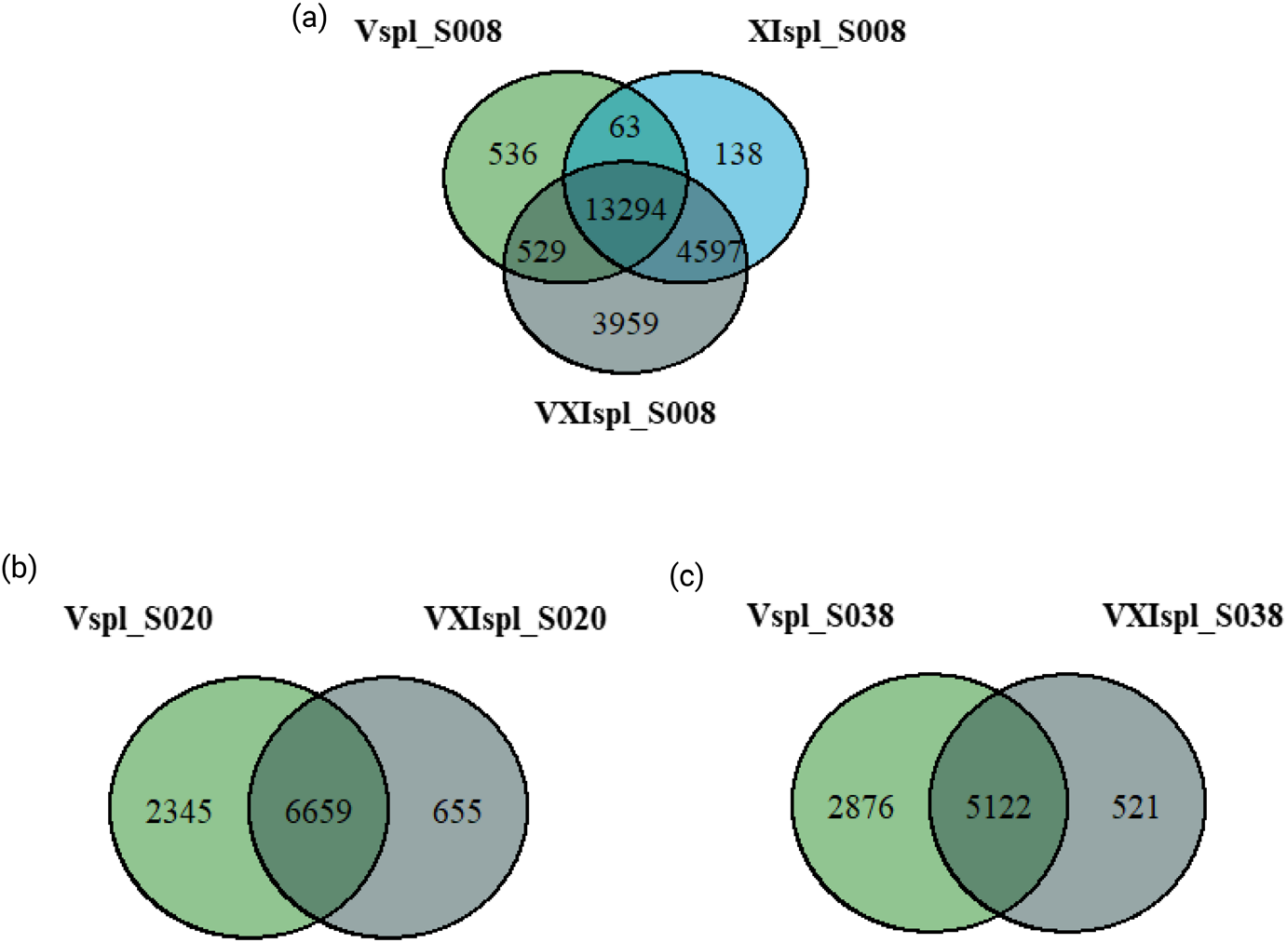
Overlap of SNPs contained in selected genomic regions between resistance comparisons. Venn diagrams show the number of SNPs located in the three main candidate regions obtained by local *C*_2_ score shared or specific to each resistance comparison on (a) scaffold S008, (b) scaffold S020, and (c) scaffold S038. Comparisons include *GpaV*_*spl*_ (Vs_SM1 and Vs_SM2 vs. 0Q_SM1 and 0Q_SM2), *GpaXI*_*spl*_ (XIs_SM1 and XIs_SM2 vs. 0Q_SM1 and 0Q_SM2), and *GpaV + XI*_*spl*_ (VXIs_SM3 and VXIs_SM4 vs. 0Q_SM1 and 0Q_SM2).

To assess the overlap of candidate SNPs between the three resistance comparisons, Venn diagrams were generated for each of the three main scaffolds identified by local C2 score analysis (Figure 4). On S008 (Figure 4a), a large number of SNPs were shared by all three comparisons (13,294), indicating significant overlap between the genomic regions associated with each resistance. Regarding the comparison between *GpaV*_*spl*_ and *GpaV + XI*_*spl*_, 8556 SNPs (i.e., 3959 + 4597, Figure 4a) were specific to *GpaV + XI*_*spl*_, whereas regarding the comparison between *GpaXI*_*spl*_ and *GpaV + XI*_*spl*_, 4488 SNPs (i.e., 3959 + 529, Figure 4a) were specific to *GpaV + XI*_*spl*_. On S020 (Figure 4b), the majority of SNPs were shared between *GpaV*_*spl*_ and *GpaV + XI*_*spl*_ (6659), with only 655 SNPs specific to *GpaV + XI*_*spl*_. On S038 (Figure 4c), a similar pattern was observed, with a large number of SNPs shared between *GpaV*_*spl*_ and *GpaV + XI*_*spl*_ (5122), and only 521 SNPs specific to *GpaV + XI*_*spl*_.

### 3.5 Evolutionary Stepping Stone

**FIGURE 5:**
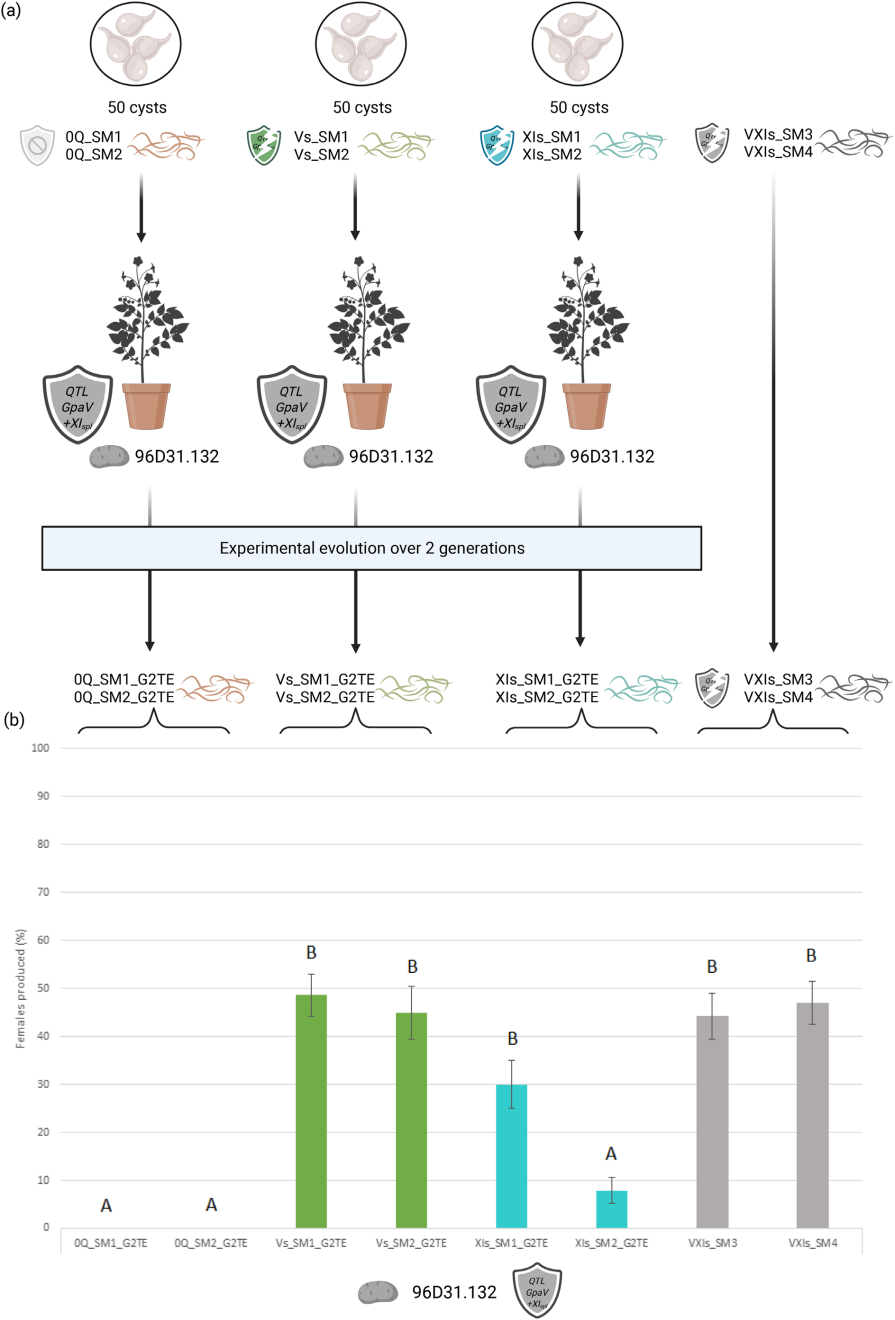
(a) Experimental evolution generations and phenotyping to test the evolutionary stepping stone hypothesis. Six *G. pallida* lineages from 50 cysts of the tenth generation of the previous experiment were reared for two generations on the *GpaV + XI*_*spl*_ genotype (96D31.132). These were susceptible background control lineages (0Q_SM1 and 0Q_SM2), *GpaV*_*spl*_ adapted lineages (Vs_SM1 and Vs_SM2) and *GpaXI*_*spl*_ adapted lineages (XIs_SM1 and XIs_SM2). (b) The lineages tested correspond to two generations on *GpaV + XI*_*spl*_ (G2TE) from lineages previously bred on susceptible background (0Q_SM1_G2TE and 0Q_SM2_G2TE), lineages previously adapted to *GpaV*_*spl*_ (Vs_SM1 and Vs_SM2) and lineages previously adapted to *GpaXI*_*spl*_ (XIs_SM1 and XIs_SM2). Letters indicate statistically significant differences between groups (ANOVA, *F*_7,103_ = 17.83, *p* < 0.0001).

Genome scan analyses revealed that adaptation to *GpaV + XI*_*spl*_ pyramidal resistance shares a high proportion of selected SNPs with adaptation to *GpaV*_*spl*_ alone, while *GpaXI*_*spl*_ shares fewer SNPs with the pyramidal combination. These results raised the question of whether prior adaptation to individual QTLs and the overlap of genomic regions shared with GpaV+XI could influence the evolutionary trajectory towards overcoming pyramidal resistance. To answer this question, a short experimental evolution was conducted to test the hypothesis that prior adaptation to *GpaV*_*spl*_ or *GpaXI*_*spl*_ could act as an evolutionary stepping stone towards adaptation to *GpaV + XI*_*spl*_. Lineages from the tenth generation of the previous experimental evolution, comprising *GpaV*_*spl*_ adapted lineages (Vs_SM1, Vs_SM2), *GpaXI*_*spl*_ adapted lineages (XIs_SM1, XIs_SM2) and control lineages (0Q_SM1, 0Q_SM2), were reared over two generations on the genotype 96D31.132 harboring the *GpaV + XI*_*spl*_ combination (Figure 5a). Starting the experimental evolution at 50 cysts, all lineages were maintained after the two generations, although the number of cysts obtained in each generation varied considerably (Figure S1). The average number of cysts obtained in G1 was 7 for 0Q lineages, 445 for Vs lineages and 12 for XIs lineages. The final number of cysts obtained in G2 was 4 for 0Q, 473 for Vs and 26 for XIs.

After two generations on the combined resistance genotype, the virulence of each G2TE lineage was quantified by assessing the percentage of females produced on 96D31.132 (*GpaV + XI*_*spl*_). The control lineages (0Q_SM1_G2TE, 0Q_SM2_G2TE) produced no females. In contrast, lineages initially adapted to *GpaV*_*spl*_ (Vs_SM1_G2TE, Vs_SM2_G2TE) produced on average 46.79% females, statistically identical to the percentage produced by *GpaV + XI*_*spl*_ adapted lineages (on average 45.60% for VXIs_SM3 and VXIs_SM4). Regarding lineages adapted to *GpaXI*_*spl*_, one lineage succeeded in adapting to the pyramided resistance (XIs_SM1_G2TE produced 30% of females) while the other did not (XIs_SM2_G2TE produced 7.87% of females). The differences between the lineages were statistically significant (*F*_7,103_ = 17.83, *p* < 0.0001) (Figure 5b).

## 4 Discussion

### 4.1 Experimental Evolution Reveals Differential Adaptation to Individual and Pyramidal Resistances

Using an experimental evolutionary approach, it was possible to compare the adaptive potential of *Globodera pallida* populations confronted with different resistance genotypes. Phenotyping of the lineages from the tenth generation of experimental evolution revealed contrasting levels of virulence depending on the resistance used. For the minor QTL *GpaXI*_*spl*_, one of the two evolved lineages produced a high proportion of females, while the other reached levels comparable to those of the susceptible control. In contrast, for the major QTL *GpaV*_*spl*_, results showed a strong difference between control and adapted lineages. The *GpaV + XI*_*spl*_ pyramidal genotype proved the most effective, with the control lineages producing no females. Moreover, a strong selection pressure was reflected in the results of experimental evolution: only 40% of the lineages initially exposed to this pyramidal resistance were maintained over 10 generations, compared with 100% for lineages selected on *GpaV*_*spl*_ or *GpaXI*_*spl*_ alone. Accordingly, it has been shown in the literature that selecting for virulence is generally rather easy in cyst nematodes, even if it depends on the population used (Dong and Opperman 1997 for *Heterodera glycines* ; Fournet et al. 2013 for *G. pallida* ; Muller 1998 for *Heterodera schachtii* ; Phillips and Blok 2008 for *G. pallida* ). But selecting with a pyramid can be much more difficult, given its reputation for providing more durable resistance than individual QTLs (Caromel et al. 2005; Djian‐Caporalino et al. 2014). These results are in accordance with theoretical expectations: the combination of several resistance factors imposes stronger selective constraints and requires more genomic modifications to adapt to these factors. However the strict sexual reproduction of cyst nematodes could facilitate, via recombination, the assembly of virulence alleles against pyramidal resistances (McDonald and Linde 2002). The overcoming can also be facilitated when virulence alleles conferring adaptation to single QTLs are already present in the population, as also observed by Mugniery et al. (2007) in their study on the heritability of virulence to different QTLs, including *GpaV*_*spl*_.

However, several limitations need to be recognized, as our conclusions are based on experimental evolution performed under controlled conditions. Consequently, we are uncertain how wild virulent lineages would behave in the face of these resistances. The absence of field validation means that the plants were grown exclusively under greenhouse conditions and may not fully reflect the complexity of field environments. In experimental evolution approaches, gene flow is null, whereas in natural populations gene flow could introduce novel beneficial alleles facilitating adaptation. Additionally, it is important to note that our findings are based on a specific avirulent population (from St‐Malo); different results might have been obtained with another initial population.

### 4.2 Genomic Basis of Adaptation Highlights Shared and Specific Signatures Across Resistance Types

To determine whether genomic data could explain the observed patterns of adaptation, genome scan analyses were carried out to identify regions subject to selection in each resistance comparison. For adaptation to *GpaV + XI*_*spl*_ pyramidal resistance, three distinct genomic regions subject to strong selection were detected on scaffolds S008, S020 and S038. These same regions were also found in the *GpaV*_*spl*_ comparison, although the signal on S008 was weaker, whereas *GpaXI*_*spl*_ showed only one weaker region on S008. The overlap of SNPs and genes in the selected regions provides an additional element of response (Figure 4 and Figure S1). On the S008 scaffold, 58% of SNPs and 57% of genes identified in the *GpaV + XI*_*spl*_ comparison were shared with the *GpaV*_*spl*_ and *GpaXI*_*spl*_ comparisons, suggesting a potential but incomplete preadaptation. Interestingly, 17% of SNPs and 15% of genes were specific to the *GpaV + XI*_*spl*_ comparison, reflecting the need for additional genomic changes for adaptation to this resistance. On S020 and S038, overlap with *GpaV*_*spl*_ was significant, with respectively 69% and 60% of SNPs, and 99% and 69% of genes shared, while only 7% and 6% of SNPs were specific to *GpaV + XI*_*spl*_ (and no genes). These results support the idea that adaptation to *GpaV*_*spl*_ alone already mobilizes most of the genomic regions required for adaptation to pyramidal resistance, with the exception of additional specific variants on the S008 scaffold, which probably explains the incomplete virulence observed in preadapted lineages.

The regions identified by *C*_2_ were also found using the *X* ^*T*^ *X* statistic, which does not consider the virulence status of lineages, showing that the main selection pressure in our experimental evolution was the resistance of the potato genotypes (Figure S2). Supporting these observations, pairwise genetic differentiation *F*_*ST*_ values calculated using all SNPs revealed a clear structuring among the experimental lineages which have evolved on genetically closed potato genotypes, coming from a single cross and differing by their QTL content (Figure S3). Lineages adapted to *GpaV + XI*_*spl*_ were genetically distinct from all other lineages. Interestingly, lineages adapted to *GpaV + XI*_*spl*_ were genetically closer to lineages adapted to *GpaV*_*spl*_ than to lineages adapted to *GpaXI*_*spl*_ or to the 0QTL control lineages. This pattern reinforces the idea that adaptation to *GpaV*_*spl*_ could involve allelic changes also relevant for adaptation to *GpaV + XI*_*spl*_.

Functional annotation of candidate genes in these regions revealed known effectors (Table S1). Among these, we focused primarily on VAP1 and 1106, as they are both well‐characterized suppressors of plant immunity, and their presence in regions under selection reinforces the biological relevance of the genomic signals identified. The VAP1 gene was consistently detected in all three types of adaptation on the S008 scaffold, highlighting its potential role in virulence. Multiple gene accessions correspond to this effector family, including Gpal_D383_g07988, which was present in all three comparisons. The 1106 effector was identified in *GpaV*_*spl*_ and *GpaV + XI*_*spl*_ comparisons on the S020 scaffold, supporting its involvement in the adaptation process. Several gene accessions associated with this effector family were found in both comparisons, including Gpal_D383_g13667, Gpal_D383_g13668, Gpal_D383_g13671, Gpal_D383_g13703, and Gpal_D383_g13714. In *Globodera rostochiensis*, VAP1 has been shown to interfere with immune signaling and disrupt Pattern‐Triggered Immunity and Effector‐triggered Immunity responses in plants (Li et al. 2021; Lozano‐Torres et al. 2012, 2014; Pogorelko et al. 2020; van Steenbrugge et al. 2023). The evolutionary conservation of VAP family members and their frequent presence in nematodes reflects its likely key role in bypassing plant defenses (Wilbers et al. 2018). The 1106 effector family, also known as GLAND4, is specific to cyst nematodes and is characterized by its ability to bind DNA and suppress plant immunity (Noon et al. 2015; Thorpe et al. 2014). The VAP1 and 1106 effectors had already been previously identified in genome scan analyses that also concerned the adaptation of nematodes to plant resistance (Eoche‐Bosy et al. 2017; Kwon et al. 2024; Lechevalier et al. 2025). The recurrence of VAP1 and 1106 in selection regions, using distinct comparisons, confirms their potential involvement in adaptation mechanisms.

However, other effector families were also detected within regions under selection and may contribute to parasitism through complementary mechanisms. Multiple accessions corresponding to glutathione synthetase‐like (GSS) effectors were found across comparisons. These GS‐like effectors result from the neofunctionalisation of a housekeeping glutathione synthetase gene and have been shown to be specifically expressed in the dorsal gland and secreted into the syncytium (Lilley et al. 2018). We also identified effectors of the IA7 family, previously shown to be expressed in the subventral glands of *G. pallida* at the J2 stage (Grenier et al. 2002; Blanchard et al. 2007). The GpIA7 effector has been demonstrated to reduce the expression of key cell cycle regulators in host plants and stimulate the endocycle when syncytium formation is triggered (Coke et al. 2021).

Overall, while the VAP1 and 1106 genes have been highlighted due to their direct and well‐established involvement in immune suppression, the co‐occurrence of other types of effectors such as GSS and IA7 effectors indicates that adaptation to resistance likely relies on multiple complementary effector functions. However, the interpretation of these results is limited by the high number of candidate genes lacking functional annotation (NA), that could correspond to effectors. Many SNPs associated with virulence map to these unannotated genes, which constrains our understanding of the full set of mechanisms involved in adaptation.

### 4.3 Prior Adaptation to Individual QTLs as Evolutionary Stepping Stones to Overcome Pyramidal Resistance

In the process of experimental evolution, adaptation to *GpaV + XI*_*spl*_ pyramidal resistance from the initial population proved particularly complicated. However, phenotyping of prior adapted lineages revealed that partial adaptation to *GpaV + XI*_*spl*_ was possible when starting from lineages already adapted to *GpaV*_*spl*_ or *GpaXI*_*spl*_ but with a different degree of adaptation.

Moreover, after two generations on *GpaV + XI*_*spl*_, lineages previously adapted to *GpaV*_*spl*_ rapidly reached a level of virulence comparable to the lineages having directly evolved on the pyramidal genotype, both in terms of phenotyping and the number of cysts produced in each generation (Figure S4). In contrast, lineages previously adapted to *GpaXI*_*spl*_ showed more variable and generally weaker responses, with one lineage successfully adapting, while the other only partially succeeded. This difference between two independent replicated lineages illustrates that the deterministic process (selection) could be slowed down by stochastic processes (genetic drift), and that the intensity of genetic drift can be strong (i.e., small effective population size) in *G. pallida* (Montarry et al. 2019). The XIs_SM1_G2TE lineage that adapted most effectively to the pyramidal genotype was not the most virulent during initial evolution on *GpaXI*_*spl*_, suggesting that the ability to switch to a new resistance background does not depend solely on the initial level of adaptation.

**FIGURE 6:**
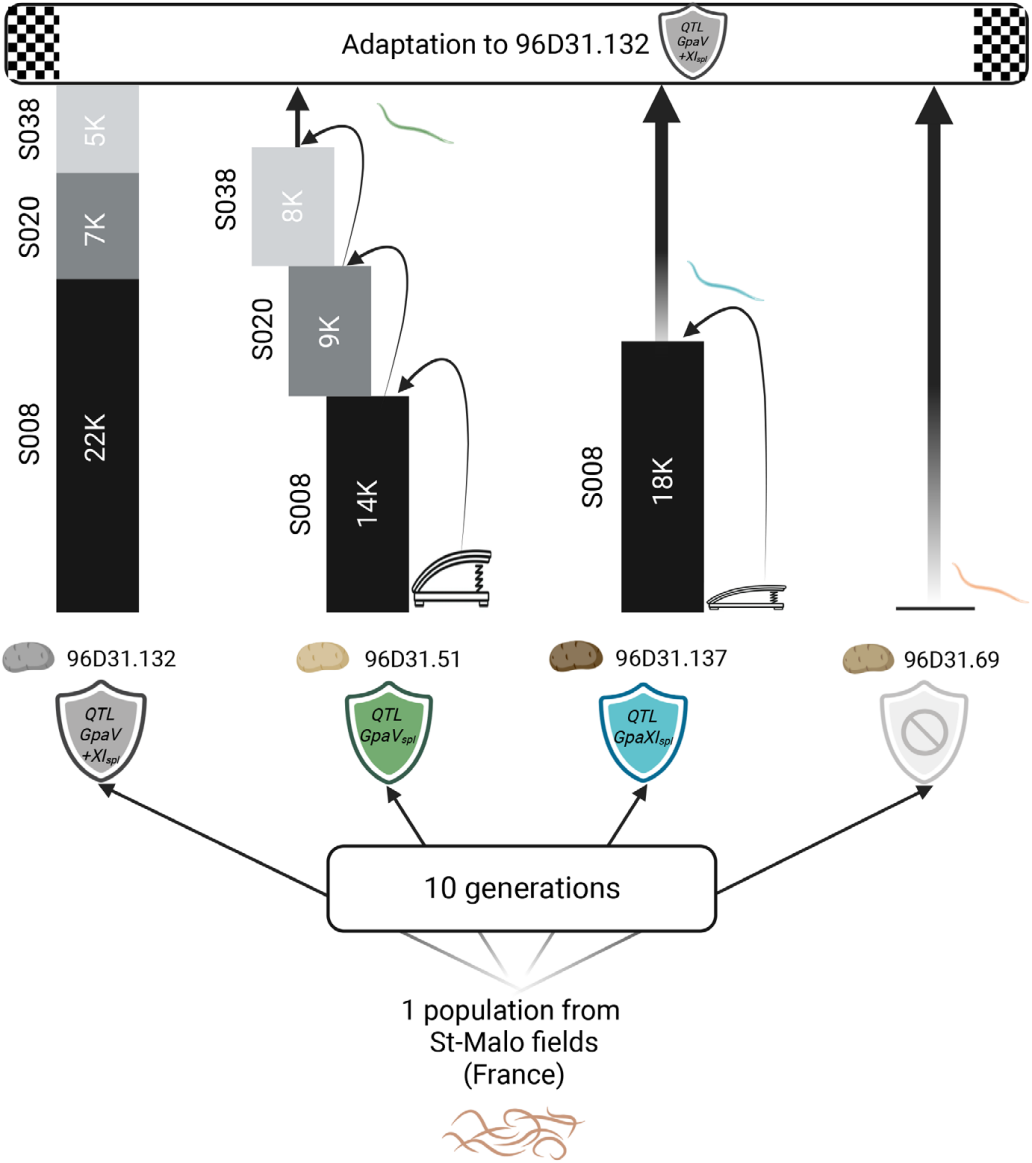
Evolutionary stepping stone to adapt to *GpaV + XI*_*spl*_ after 10 generations of experimental evolution on the susceptible genotype, *GpaV*_*spl*_ or *GpaXI*_*spl*_. For each resistance, the number of SNPs identified in the regions under selection (identified by the local score based on *C*_2_ statistics) is shown.

These results are consistent with genomic analyses, which revealed greater overlap in selected regions and SNPs content between *GpaV*_*spl*_ and *GpaV + XI*_*spl*_ than between *GpaXI*_*spl*_ and *GpaV + XI*_*spl*_. This suggests that the adaptation of *GpaV*_*spl*_ to pyramidal resistance requires fewer additional changes than that of *GpaXI*_*spl*_. *GpaV*_*spl*_ adapted lineages already carry a large number of SNPs also present in *GpaV + XI*_*spl*_, while *GpaXI*_*spl*_ adapted lineages lack a larger number (Figure 6). From a theoretical point of view, our last experimental evolution during two generations on the potato genotype with *GpaV + XI*_*spl*_ could be considered as an “historical difference experiment” in which previously diverged populations evolve under identical conditions (Blount et al. 2018). In this context our result could be seen as an example of “historical contingency” as it showed that the outcome of evolution (the level of adaptation to *GpaV + XI*_*spl*_) may be affected by the history of the lineages (previously adapted to either *GpaV*_*spl*_ or *GpaXI*_*spl*_). Both histories worked as stepping stones, but in this particular adaptive landscape, the path is clearly faster when the nematode is prior adapted to *GpaV*_*spl*_. As our results suggested that prior adaptation to individual QTLs may facilitate the evolutionary trajectory towards overcoming pyramidal resistance, this opens the way for predictive approaches to resistance deployment.

### 4.4 Implications for Resistance Deployment Strategies

Our results have several potential implications for the deployment of resistance in the field, not least if we consider that pyramidal resistance is seen as a means of improving the durability of resistance genes (Mundt 2018). The use of *GpaXI*_*spl*_ alone offered limited protection, as shown by the phenotypic and genomic data, and is therefore not recommended. *GpaV*_*spl*_ alone offers better protection, although its durability seems compromised if deployed alone for long periods. In addition to these limitations, the deployment of individual QTLs prior to pyramidal combination could compromise their durability.

One possible strategy for maintaining resistance durability would be to alternate *GpaV*_*spl*_ or *GpaV + XI*_*spl*_ with other resistances such as *GpaV*_*vrn*_ which trigger distinct virulence mechanisms and do not exhibit cross‐virulence (Fournet et al. 2013; Lechevalier et al. 2025). This could delay the accumulation of adaptive alleles and increase the durability of resistance. Yet this proposed absence of cross‐virulence is based solely on experimental evidence and has not been validated under field conditions, where interactions may be more complex. This highlights the importance of a cautious and well‐coordinated approach to resistance deployment. In particular, deploying only the pyramidal resistance (*GpaV + XI*_*spl*_), that is, without using the individual components, would require strong collaboration between the different breeder companies and to monitor carefully the QTLs present in commercial varieties. This type of collaborative management of the available resistances is also relevant for resistances in other crops, for example, to fungal pathogens in rapeseed and grapevine (Balesdent et al. 2022; Bettinelli et al. 2023).

Such coordination would also facilitate the integration of predictive tools into breeding programmes. The observation of preferential adaptive pathways supports the value of predictive genomics for resistance deployment: by identifying shared genomic signatures, it becomes possible to anticipate the risk of pyramidal resistance breakdown. In this context, increasing the diversity of resistance sources and monitoring virulence alleles in nematode populations will be essential to develop robust long‐term strategies. This is consistent with the idea that sustainable resistance management requires more research into population genetics (Saubin et al. 2023). Overall, our results argue in favour of management approaches that combine resistance design, field deployment strategies and prediction of the evolution of virulence alleles in populations.

## Conflicts of Interest

The authors declare no conflicts of interest.

## Supporting information


**Data S1:** eva70181‐sup‐0001‐Supinfo.docx.

**Table S1:** Table of candidate genes in regions under selection that have passed all filtering stages. Each sheet corresponds to the content of the candidate region of a scaffold associated with a specific resistance. For each gene is associated: the identifier (GENE_ID), the functional annotation (ANNOTATION), the number of predicted transmembrane domains (TM), and the presence or absence of a predicted signal peptide (SP, 1 indicating presence and 0 absence).

## Data Availability

Raw reads of the 8 samples are available at the European Nucleotide Archive database system (https://www.ebi.ac.uk/ena/browser/home) under the sample accession numbers ERS25839542 to ERS25839547 (project accession number PRJEB96172), ERS25052903 and ERS25052904 (project accession number PRJEB90550).
