# Supplementary material for: Adaptation of Globodera pallida to Individual Components Compromises the Durability of Pyramided Resistance in Potato
Source: Evol Appl. 2025 Nov 18;18(11):e70181. doi: 10.1111/eva.70181 (PMC12626769; doi:10.1111/eva.70181)
Supplement: Supplementary file 1 — Data S1: eva70181‐sup‐0001‐Supinfo.docx. [file EVA-18-e70181-s002.docx]

**METHODS S1**

**Plant material and experimental evolution design**

The experimental evolution was conducted in controlled greenhouse conditions over ten consecutive years, each corresponding to one generation of *Globodera pallida*. Lineages originating form a natural population from Saint-Malo, France (SM) were reared independently on each potato genotype: 96D31.69 (susceptible, no resistance QTL), 96D31.51 (harbouring *GpaV_spl_*), 96D31.137 (harbouring *GpaXI_spl_*), and 96D31.132 (harbouring both *GpaV_spl_* and *GpaXI_spl_*). Generations one to three were conducted in ten litter pots containing three potato tubers per pot. At the end of generation three, cysts were extracted using a Kort elutriator and placed in a single tulle bag to perform the next generation in two litter pots. From generation five to generation ten, the experiment was standardized in one litter pots, each containing a single potato plant. The number of cysts used for inoculation varied depending on the output of the previous generation, ranging from 143 to 1000 cysts per pot. Since the eight generation, inoculation was normalized to 200 cysts per pot.

To determine whether adaptation to resistance to a single QTL (*GpaV_spl_* or *GpaXI_spl_*) could serve as an evolutionary stepping stone for overcoming the pyramided resistance (*GpaV+XI_spl_*), a short experimental evolution was performed. For each G10 lineage evolved on 96D31.69 (no resistance QTL), 96D31.51 (*GpaV_spl_*) and 96D31.137 (*GpaXI_spl_*), 50 cysts were placed in tulle bags and inoculated onto a 96D31.132 (*GpaV+XI_spl_*) tuber in 1L pots. After one generation, the cysts were extracted using a Kort elutriator and one to 50 (depending on availability, see Fig. S1) of them were used to reinoculated the same genotype to obtain the second generation.

**Phenotyping**

For all phenotyping experiments, hatching of second-stage juveniles (J2) was stimulated using root exudates from the susceptible cv. Désirée. For each lineage, a minimum of 20 cysts were used, placed on 250 µm sieves and continuously exposed to exudates for 13 days. Batches of ten newly hatched J2 were inoculated onto individual root apex grown in Petri dishes, with a maximum of one potato tuber per dish. Depending on the genotype, 10 to 20 roots were inoculated per lineage. After 26 days, roots were dissected using a binocular magnifying glass to count the number of females.

Statistical analyses of the phenotyping were performed using the R software (v4.2.2). A linear model was fitted to the data to assess the effect on the number of females produced. The validity of the analyses was verified by checking the underlying assumptions of the model. The independence, normality and homogeneity of residuals were checked by Durbin-Watson, Shapiro and Levene tests, respectively. An analysis of variance (ANOVA) was performed for each potato cultivar to assess the effect of lineages on the number of females produced. In the event of a significant difference (p < 0.05), a Tukey test was applied to compare means between lineages.

**Extraction, library preparation and sequencing**

To form each eight pools, 200 cysts of each lineage were hydrated in sterile water and individually opened. Three J2/cyst were collected to form pools of 600 J2 for each lineage, which were stored in 1.5 mL tubes at -80°C until DNA extraction.

DNA Prep libraries were prepared according to the Illumina protocol DNA Prep, (M) Tagmentation (96 samples, IPB, 20060059). DNA was fragmented by tagmentation with bead-linked transposomes from the library kit. Size selection was performed using Illumina Purification Beads (1/8 bead/water ratio) from the library kit. Adaptators IDT for Illumina DNA/RNA UD Indexes Set ABCD, Tagmentation (96 indexes, 96 samples respectively 20027213, 20027214, 20042666, 20042667) were ligated prior to sequencing. Library quality was assessed using a fragment analyser (Agilent) with a high sensitivity NGS kit (DNF-474-0500). The average library size achieved is 500 bp.

**Processing of Pool seq data**

Quality control and filtering of raw reads was performed using *FastQC* v0.11.7 (Andrews, 2015) and *fastp* v0.20.0 (Chen et al., 2018) to eliminate low-quality bases (Phred-quality score < 30) and residual adapters. Filtered reads were mapped against the *Globodera pallida* D383 reference genome (van Steenbrugge et al., 2023) using *mem* algorithm from *BWA* v0.7.17 (Li, 2013) with default settings. This reference genome, with a total size of 113 Mb, is assembled into 163 scaffolds (N50 = 2.9 Mb). The resulting BAM files were sorted and duplicates removed using *Picard* tool v2.18.2 (broadinstitute.github.io/picard/). Reads not properly aligned were discarded with *Samtools* v1.6 (Li et al., 2009) and statistics associated with BAM files were calculated with *flagstat* and *coverage* commands.

Variant calling was performed using the haplotype caller implemented in *FreeBayes* v1.1.0 (Garrison & Marth, 2012) with the following options -K -C 1 -F 0.01 -G 5 -n 4 -m 30 -q 20. Note that some of the multi-nucleotide polymorphisms (MNPs) generated by adjacent (bi-allelic) SNPs that were called under these settings were further atomized using a custom awk script. The resulting vcf file was parsed with the *vcf2pooldata* function of the R package *poolfstat* (v2.2.0) (Gautier et al., 2022) with options min.maf=0.01, min.cov.per.pool=20 and max.cov.per.pool=400.

**Detection and content of regions involved in adaptations**

Pairwise *F*_ST_ were estimated with the *computeFST* function of *poolfstat*. As recommended in the *BayPass* manual, SNPs were subsampled using the *pooldata2genobaypass* function. Specifically, 31 subsamples of around 75,000 SNPs were generated by selecting one SNP every 31 sites along the genome. The sub-datasets obtained were analyzed in parallel with *BayPass* v2.41 (Gautier, 2015) to detect genomic signatures of adaptive differentiation (based on the XtX statistics) and to perform analysis of association with the virulence/avirulence status (based on the C_2_ contrast statistic). To evaluate reproducibility of the results, each analysis was independently repeated three times specifying different seed for the Random Number Generator (-seed option). The *X^T^X* statistic, was estimated from the core model and measures an increase in allelic frequency differentiation compared with expectations under a neutral model, while accounting for the covariance structure resulting from shared population history (Günther & Coop, 2013). An analysis with the C_2_ statistic (under the core model) was carried out to compare standardized population allele frequencies between the two population groups defined by a binary covariate determining the virulence status (-1 = avirulent, 1 = virulent) (Olazcuaga et al., 2020). This analysis enabled us to identify the loci for which changes in allele frequency observed with XtX were significantly associated with the virulence phenotype.

To improve the detection of selection signals and the delineation of differentiated or associated genomic windows, a local score was applied to both *X^T^X* and C_2_ statistics, using the method described by Fariello et al. (2017) as implemented in the compute.local.scores R function available in the BayPass software package (v3.0) ran with default options. This approach makes it possible to identify genomic regions subject to selection by aggregating information from the statistics calculated for each individual marker. By applying this score, significant signals are amplified when they are consistent across several neighbouring positions, improving the robustness of the results and facilitating the identification of regions subject to selection.

The results of three independent analyses were merged for each type of local score analysis (*X^T^X* or C_2_) to retain only the SNPs common to all three and appearing in the signals of the significant regions of *X^T^X* and C_2_.

These merged sets of SNPs were then used to perform a correlation analysis between allele frequency and the virulence levels obtained through phenotyping. For each comparison (96d31.69 vs. 96d31.51 or 96D31.137 vs. 96D31.132), the correlation between allele frequency and the average percentage of females observed on the corresponding resistant potato was tested independently for each lineage. A linear regression model was applied to evaluate this relationship using R software (v4.2.2). SNPs with a p-value < 0.05 and an R² > 0.6 were retained for further analysis. This threshold ensures that correlations are significant and explain a sufficient proportion of the observed variance, thus reducing the risk of false positives. The selected SNPs were then annotated using *SnpEff* v5.2.1 (Cingolani et al., 2012) to identify the implicated genes and predict the functional impact of the variants. An analysis of secretion patterns was performed on the identified genes to detect any secreted effectors. The presence or absence of signal peptides (SP) and transmembrane domains (TM) were predicted using Phobius v1.01 (Käll et al., 2004).

**SUPPLEMENTARY FIGURES**

**Fig. S1** (a) Experimental evolution generations and phenotyping to test the evolutionary stepping stone hypothesis. Six *G. pallida* lineages from 50 cysts of the tenth generation of the previous experiment were reared for two generations on the *GpaV+XI_spl_* genotype (96D31.132). These were susceptible background control lineages (0Q_SM1 and 0Q_SM2), *GpaV_spl_* adapted lineages (Vs_SM1 and Vs_SM2) and *GpaXI_spl_* adapted lineages (XIs_SM1 and XIs_SM2).

(b) Cyst production during short experimental evolution on pyramidal resistance. Number of cysts produced after each generation (G1 and G2) during the short-term experimental evolution conducted on the *GpaV+XI_spl_* genotype (96D31.132) are indicate in blue and red respectively. The experiment involved *G. pallida* lineages previously adapted to *GpaV_spl_* (Vs_SM1 and Vs_SM2), *GpaXI_spl_* (XIs_SM1 and XIs_SM2), and control lineages from the susceptible background (0Q_SM1 and 0Q_SM2).

**Fig. S2** Overlap of candidate genes after filtering and contained in selected genomic regions between resistance comparisons. Venn diagrams show the number of genes located in the three main candidate regions obtained by local score C_2_ shared or specific to each resistance comparison on (a) scaffold S008, (b) scaffold S020, and (c) scaffold S038. Comparisons include *GpaV_spl_* (Vs_SM1 and Vs_SM2 vs. 0Q_SM1 and 0Q_SM2), *GpaXI_spl_* (XIs_SM1 and XIs_SM2 vs. 0Q_SM1 and 0Q_SM2), and *GpaV+XI_spl_* (VXIs_SM3 and VXIs_SM4 vs. 0Q_SM1 and 0Q_SM2).

**Fig. S3** Manhattan plots showing selection signatures in the *Globodera pallida* genome detected between virulent and avirulent lineages. Manhattan plots (a) and (b) were constructed from SNPs derived from the comparison between lineages reared on 96D31.69 and 96D31.132 (i.e. adaptation to *GpaV+XI_spl_*), Manhattan plots (c) to (d) from the comparison between lineages 96D31.69 and 96D31.51 (i.e. adaptation to *GpaV_spl_*), and finally Manhattan plots (e) to (f) from the comparison between lines 96D31.69 and 96D31.137 (i.e. adaptation to *GpaXI_spl_*). Scaffold names are shown on the horizontal axis, the vertical axis shows for each SNP the *X^T^X* values for (a), (c) and (e); and the local *X^T^X* score values for (b), (d) and (f). Boxes highlight scaffolds containing regions with strong indications of selection.

**Fig. S4** Neighbor joining (NJ) tree of samples based on Pairwise *F*_ST_ estimated with the *computeFST* function of *poolfstat*. The NJ includes control *G. pallida* lineages reared on the genotype without resistance QTL (0Q_SM1 and 0Q_SM2), lineages reared on *GpaV_spl_* (Vs_SM1 and Vs_SM2), lineages reared on *GpaXI_spl_* (XIs_SM1 and XIs_SM2) and lineages reared on *GpaV+XI_spl_* (VXIs_SM3 and VXIs_SM4).

**Table. S1** Table of candidate genes in regions under selection that have passed all filtering stages. Each sheet corresponds to the content of the candidate region of a scaffold associated with a specific resistance. For each gene is associated: the identifier (GENE_ID), the functional annotation (ANNOTATION), the number of predicted transmembrane domains (TM) and the presence or absence of a predicted signal peptide (SP, 1 indicating presence and 0 absence).

**Fig. S1**


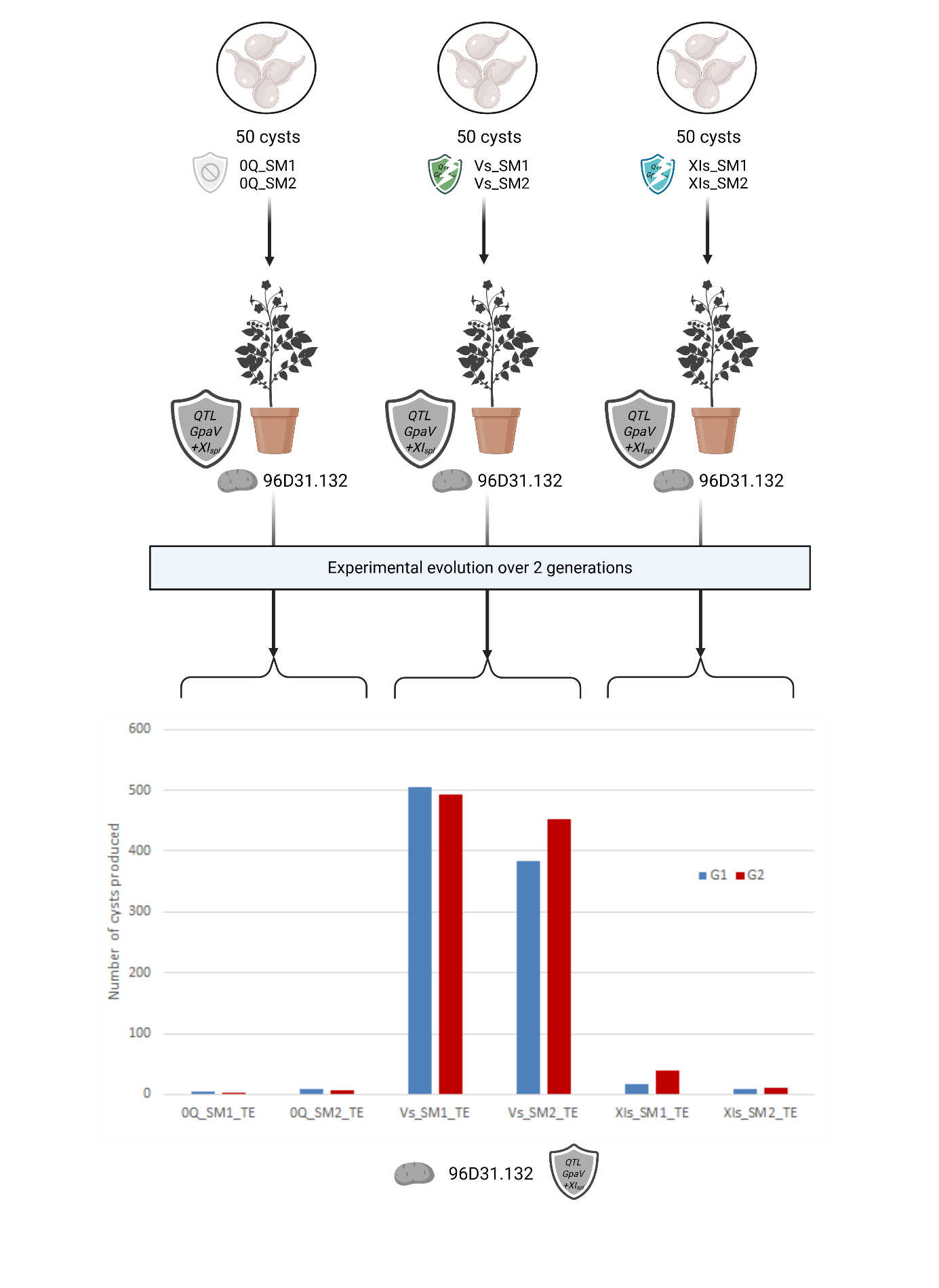


**Fig. S2**


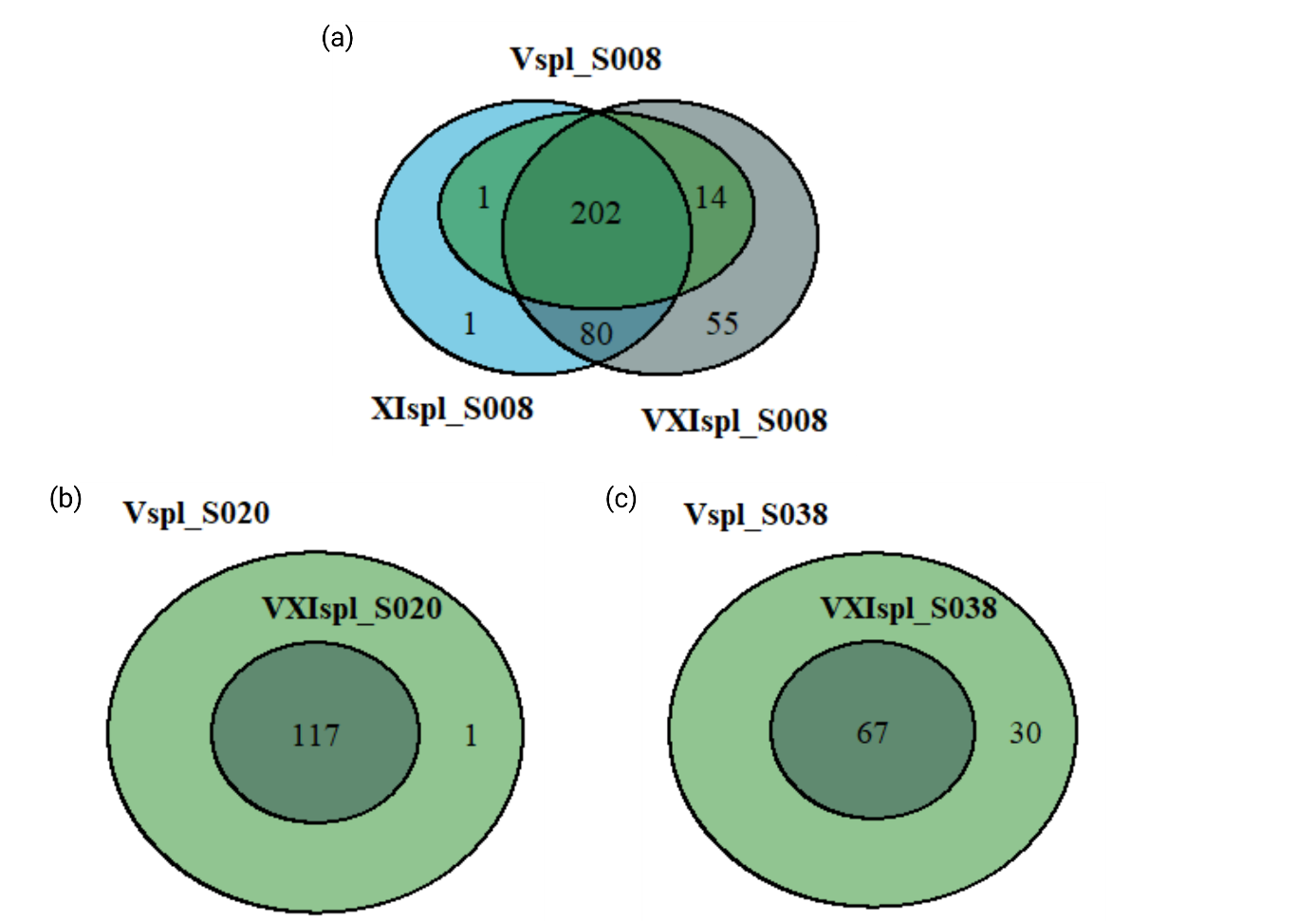


**
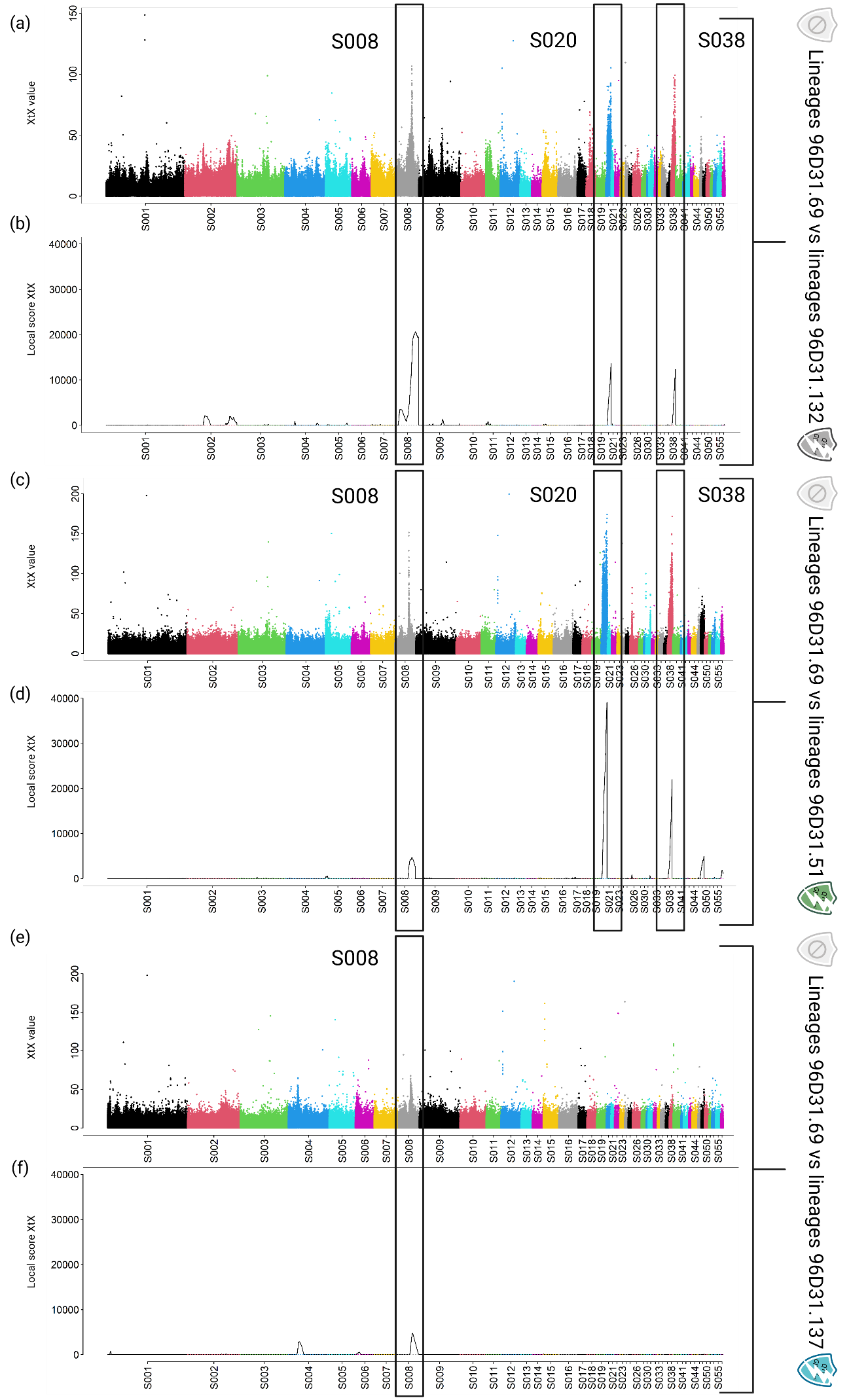
Fig. S3**

**Fig. S4**


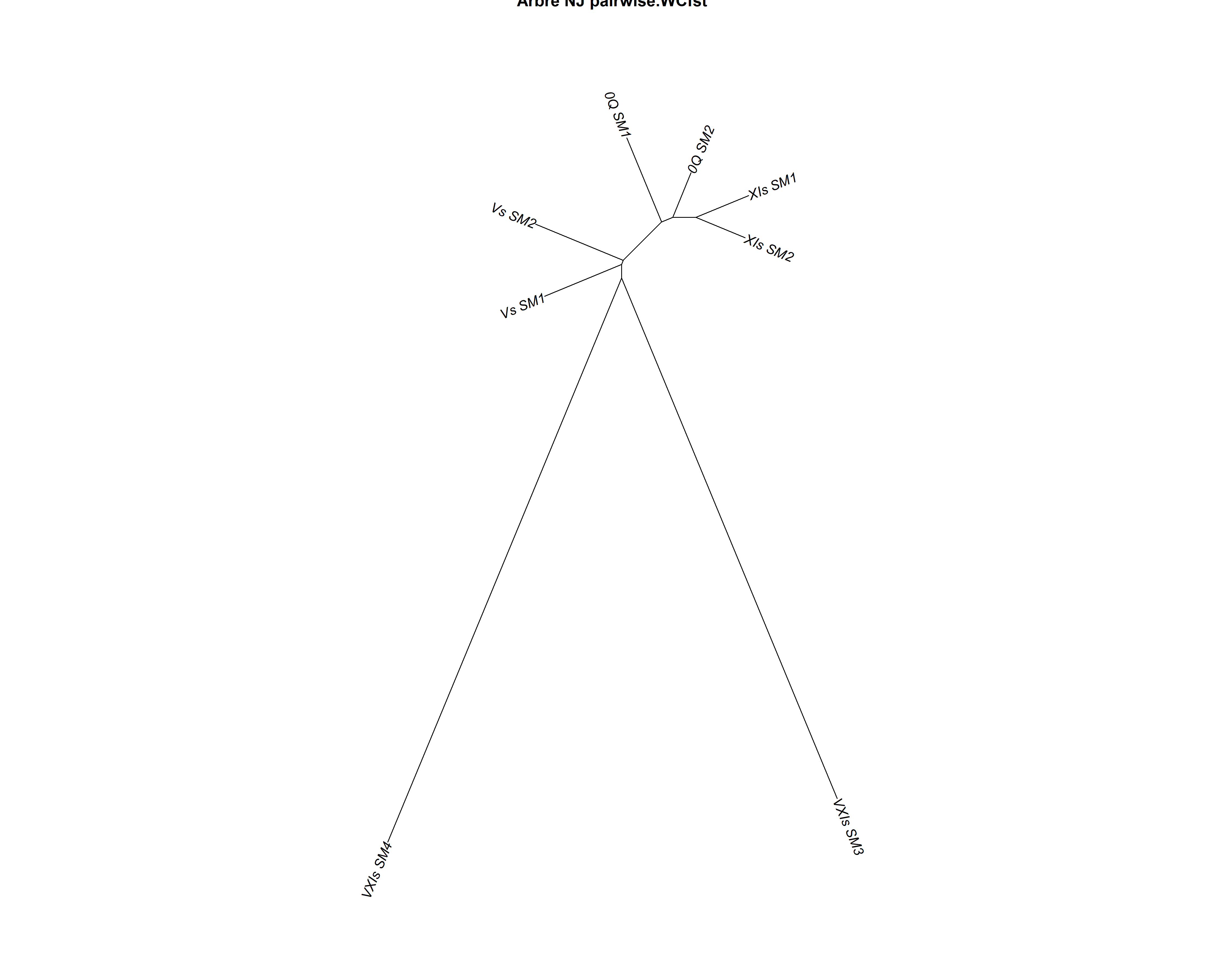


**REFERENCES**

Andrews, S. (2015). *FastQC*. https://qubeshub.org/resources/fastqc

Chen, S., Zhou, Y., Chen, Y., & Gu, J. (2018). fastp : An ultra-fast all-in-one FASTQ preprocessor. *Bioinformatics*, *34*(17), i884‑i890. https://doi.org/10.1093/bioinformatics/bty560

Cingolani, P., Platts, A., Wang, L. L., Coon, M., Nguyen, T., Wang, L., Land, S. J., Lu, X., & Ruden, D. M. (2012). A program for annotating and predicting the effects of single nucleotide polymorphisms, SnpEff: SNPs in the genome of *Drosophila melanogaster* strain w ^1118^ ; iso-2; iso-3. *Fly*, *6*(2), 80-92. https://doi.org/10.4161/fly.19695

Fariello, M. I., Boitard, S., Mercier, S., Robelin, D., Faraut, T., Arnould, C., Recoquillay, J., Bouchez, O., Salin, G., Dehais, P., Gourichon, D., Leroux, S., Pitel, F., Leterrier, C., & SanCristobal, M. (2017). Accounting for linkage disequilibrium in genome scans for selection without individual genotypes : The local score approach. *Molecular Ecology*, *26*(14), 3700‑3714. https://doi.org/10.1111/mec.14141

Garrison, E., & Marth, G. (2012). Haplotype-based variant detection from short-read sequencing (arXiv:1207.3907). arXiv. https://doi.org/10.48550/arXiv.1207.3907

Gautier, M. (2015). Genome-wide scan for adaptive divergence and association with population-specific covariates. *Genetics*, *201*(4), 1555–1579. https://doi.org/10.1534/genetics.115.181453

Gautier, M., Vitalis, R., Flori, L., & Estoup, A. (2022). F-Statistics estimation and admixture graph construction with Pool-Seq or allele count data using the R package poolfstat. *Molecular Ecology Resources*, *22*(4), 1394‑1416. https://doi.org/10.1111/1755-0998.13557

Günther, T., & Coop, G. (2013). Robust identification of local adaptation from allele frequencies. *Genetics*, *195*(1), 205–220. https://doi.org/10.1534/genetics.113.152462

Käll, L., Krogh, A., & Sonnhammer, E. L. L. (2004). A combined transmembrane topology and signal peptide prediction method. *Journal of Molecular Biology*, *338*(5), 1027–1036. https://doi.org/10.1016/j.jmb.2004.03.016

Li, H. (2013). Aligning sequence reads, clone sequences and assembly contigs with BWA-MEM. arXiv, 1303-3997. https://doi.org/10.48550/arXiv.1303.3997

Li, H., Handsaker, B., Wysoker, A., Fennell, T., Ruan, J., Homer, N., Marth, G., Abecasis, G., Durbin, R., & 1000 Genome Project Data Processing Subgroup. (2009). The sequence alignment/map format and SAMtools. *Bioinformatics*, *25*(16), 2078–2079. https://doi.org/10.1093/bioinformatics/btp352

Olazcuaga, L., Loiseau, A., Parrinello, H., Paris, M., Fraimout, A., Guedot, C., Diepenbrock, L. M., Kenis, M., Zhang, J., Chen, X., Borowiec, N., Facon, B., Vogt, H., Price, D. K., Vogel, H., Prud’homme, B., Estoup, A., & Gautier, M. (2020). A whole-genome scan for association with invasion success in the fruit fly *Drosophila suzukii* using contrasts of allele frequencies corrected for population structure. *Molecular Biology and Evolution*, *37*(8), 2369–2385. https://doi.org/10.1093/molbev/msaa098

van Steenbrugge, J. J. M., van den Elsen, S., Holterman, M., Lozano-Torres, J. L., Putker, V., Thorpe, P., Goverse, A., Sterken, M. G., Smant, G., & Helder, J. (2023). Comparative genomics among cyst nematodes reveals distinct evolutionary histories among effector families and an irregular distribution of effector-associated promoter motifs. *Molecular Ecology*, *32*(6), 1515‑1529. https://doi.org/10.1111/mec.16505
